# CD4 T cell activation by B cells in human *Leishmania (Viannia)* infection

**DOI:** 10.1186/1471-2334-14-108

**Published:** 2014-02-25

**Authors:** Daniel Rodriguez-Pinto, Nancy Gore Saravia, Diane McMahon-Pratt

**Affiliations:** 1 Centro Internacional de Entrenamiento e Investigaciones Médicas (CIDEIM), Cali, Colombia; 2 Yale University School of Public Health, New Haven, CT, USA; 3 Current Address: Facultad de Medicina, Facultad de Ciencias de la Salud, Universidad de las Américas, Quito, Ecuador

**Keywords:** B cells, Cutaneous leishmaniasis, *Leishmania (Viannia)*

## Abstract

**Background:**

An effective adaptive immune response requires activation of specific CD4 T cells. The capacity of B cells to activate CD4 T cells in human cutaneous leishmaniasis caused by *Leishmania (Viannia)* has not been evaluated.

**Methods:**

CD4 T cell activation by B cells of cutaneous leishmaniasis patients was evaluated by culture of PBMCs or purified B cells and CD4 T cells with *Leishmania panamensis* antigens. CD4 T cell and B cell activation markers were evaluated by flow cytometry and 13 cytokines were measured in supernatants with a bead-based capture assay. The effect of *Leishmania* antigens on BCR-mediated endocytosis of ovalbumin was evaluated in the Ramos human B cell line by targeting the antigen with anti-IgM-biotin and anti-biotin-ovalbumin-FITC.

**Results:**

Culture of PBMCs from cutaneous leishmaniasis patients with *Leishmania* antigens resulted in upregulation of the activation markers CD25 and CD69 as well as increased frequency of CD25^hi^CD127^-^ cells among CD4 T cells. Concomitantly, B cells upregulated the costimulatory molecule CD86. These changes were not observed in PBMCs from healthy subjects, indicating participation of *Leishmania*-specific lymphocytes expanded *in vivo*. Purified B cells from these patients, when interacting with purified CD4 T cells and *Leishmania* antigens, were capable of inducing significant increases in CD25 and CD69 expression and CD25^hi^CD127^-^ frequency in CD4 T cells. These changes were associated with upregulation of CD86 in B cells. Comparison of changes in CD4 T cell activation parameters between PBMC and B cell/CD4 T cell cultures showed no statistically significant differences; further, significant secretion of IFN-γ, TNF-α, IL-6 and IL-13 was induced in both types of cultures. Additionally, culture with *Leishmania* antigens enhanced BCR-mediated endocytosis of ovalbumin in Ramos human B cells.

**Conclusions:**

The capacity of B cells specific for *Leishmania* antigens in peripheral blood of cutaneous leishmaniasis patients to activate CD4 T cells and induce cytokine secretion is similar to that of all cell populations present in PBMCs. This capacity implicates B cells as a plausible target for modulation of the immune response to *Leishmania* infection as a therapeutic strategy.

## Background

Leishmaniasis is a parasitic disease endemic in 88 countries that affects an estimated 12 million people in the rural areas of the tropical and subtropical regions of the world. In South America, *Leishmania* species of the *Viannia* subgenus cause cutaneous and muco-cutaneous disease that can become chronic and cause severe disfigurement. In spite of the advances in knowledge of parasite biology and the host immune response, effective and safe treatment remains a challenge and there is yet no approved vaccine [1,2]. These needs may be addressed by manipulating the host immune response to obtain parasite elimination without tissue damage.

Professional antigen presenting cells (APCs) initiate the adaptive immune response by activating CD4 T cells. Activation of APCs, in the form of MHCII molecule upregulation and costimulatory molecule expression, is essential for induction of immunity, and the cytokines secreted during antigen presentation shape the ensuing response. In the murine model of cutaneous leishmaniasis (CL) caused by *L. major*, IL-12 secretion by APCs is crucial for generation of IFN-γ-secreting Th1 cells, macrophage activation and parasite elimination [3]. Failure to generate a Th1 response leads to chronic disease. In the human disease caused by *L. (Viannia)*, cells from patients with chronic disease secrete large amounts of IFN-γ and TNF-α but also Th2 and regulatory cytokines such as IL-13 and IL-10, resulting in an unbalanced immune response, excessive inflammation, tissue damage, and parasite survival [4-8]. A similar picture was observed in a murine model of chronic *L. (V.) panamensis* infection where IL-13 was shown to be crucial for development of pathology [4]. Since CD4 T cell activation by APCs leads to this harmful response, modulation of this event could promote healing or prevent disease.

Three types of APCs are recognized: dendritic cells (DCs), macrophages and B cells. As the natural host of *Leishmania*, macrophages would be the ideal candidates for initiating the adaptive immune response in CL. However, it is well documented that infection of macrophages with *Leishmania* does not induce MHCII molecule upregulation, costimulatory molecule expression or IL-12 secretion, but rather inhibits these processes, shutting down antigen presentation by macrophages. These effects have been shown in a range of *Leishmania* species in both animal models and human cells [9-12]. On the other hand, DC function has been more difficult to determine, as both activation and inhibition of APC function have been found. In the murine *L. major* model it is well recognized that DCs initiate the immune response and secrete IL-12 in the resistant phenotype [13]. However, studies with other *Leishmania* species have shown that infection with parasites does not lead to DC activation [14-18]. Notably, *L. (V.) braziliensis* infection of DCs inhibits cell activation and antigen presentation while uninfected neighboring DCs are able to upregulate MHCII and costimulatory molecules and induce T cell activation [18]. Thus, it seems that induction of immunity by DCs in CL depends on their avoidance of infection. In summary, both macrophage and DC APC function can be inhibited by *L. (Viannia)*. Therefore, the participation of B cells in CD4 T cell activation in CL caused by species of this subgenus may be crucial.

The role of B cells in human CL caused by *L. (Viannia)* has not been defined. Histological studies in Colombian patients infected with *L. (Viannia)* have revealed prominent B cell infiltration of skin lesions and leishmanin skin test reaction sites [19,20], and a study from Brazil showed a significant increase in B cell frequency in lymph node aspirates of patients that presented lymphadenopathies associated with the late stage of lesion development [21]. These findings suggest that B cells may play an important role in the immune response to *L. (Viannia)*. In mice, lesion development caused by *L. panamensis* was found to be delayed in the absence of B cells, although final lesion size and parasite load were not affected [4]. B cells have been shown in murine models of leishmaniasis to contribute to immunologic regulation through production of cytokines and immunoglobulins and as a result of antigen presentation [22-29]. However, variation occurs that may depend upon the species, specific *Leishmania* strain and mouse genetics [27,29].

The contribution of B cells in CD4 T cell activation in human CL or its pathogenesis is yet unexplored. B cells specific for a particular antigen have an exquisite capacity to concentrate and direct the antigen to the appropriate processing compartments. Furthermore, BCR signaling induces optimal APC activation, making B cells competent APCs capable of activating naïve CD4 T cells. Hence, the participation of B cells as APCs has been shown to be essential in many different contexts [30-35]. Furthermore, their capacity to secrete several cytokines capable of inducing Th1, Th2 and regulatory responses has been well documented [36]. For these reasons, and in order to identify immunomodulatory intervention strategies we have examined the capacity of B cells to activate CD4 T cells in CL.

In the present study, CD4 T cell activation by B cells was assessed in cells from patients with CL caused by *L. (Viannia)* species. We found that B cells upregulated the costimulatory molecule CD86 in PBMC cultures stimulated with *L. panamensis* and were able to activate CD4 T cells and induce cytokine secretion in the absence of other recognized APCs. Furthermore, we show that exposure to *L. panamensis* antigen enhances BCR-mediated endocytosis by the Ramos human B cell line. Taken together these results indicate a role for B cells in the modulating the immune response to *L. panamensis*.

## Methods

### Study design

We evaluated the response of cells from CL patients from the southwestern region of Colombia where most cases are caused by *L. panamensis* or *L. braziliensis*. We have previously shown that there is a strong recall response to *L. panamensis* antigens in patients from this region regardless of the infecting species [37-39]. Thus, we stimulated cells with promastigote *L. panamensis* antigen (pLAg) obtained from *L. panamensis* strain MHOM/COL/81/L13. First, we compared CD4 T cell and APC activation in CL patients and healthy subjects by culturing PBMCs with pLAg and measuring CD4 T cell, B cell and DC activation markers. We then determined the competence of B cells for CD4 T cell activation by culturing them with CD4 T cells and pLAg and evaluating activation of both cell types. We evaluated the cytokine secretion profile of PBMC and B cell/CD4 T cell cultures to establish whether the cytokine profile induced by B cell-CD4 T cell interaction is similar to that induced when all PBMCs participate. Finally, we examined the effects of exposure to pLAg on a human B cell line in relation with induction of activation markers and BCR-mediated endocytosis.

### Human subjects and cells

Peripheral blood samples (60 to 100 mL) were obtained from CL patients from the southwestern region of Colombia (Departments of Valle del Cauca and Nariño). Participants included 8 males and 2 females of 20 to 65 years of age (median = 27), who had typical cutaneous lesions, were parasitologically diagnosed by smear, culture or biopsy, and had not received anti-leishmanial treatment before enrollment. Blood samples from four healthy subjects from non-endemic areas were obtained as controls. All subjects had negative serology for HIV and HTLV-1. All participants provided written informed consent. The study protocol, consent forms and all procedures were approved by the CIDEIM Institutional Review Board for the ethical conduct of research involving human subjects.

PBMCs were isolated by centrifugation over Histopaque-1077 (Sigma-Aldrich, St. Louis, MO). B cells and CD4 T cells were isolated by MACS using the B cell isolation and CD4^+^ T cell isolation kits II, respectively (Miltenyi Biotec, Bergisch-Gladbach, Germany) following the manufacturer’s instructions. Purity assessed by staining with anti-CD20 or anti-CD4 was ≥95% for both populations. Ramos cells were obtained from the American Type Culture Collection (Rockville, MD).

### Cell culture

pLAg was prepared by suspending promastigotes of the *L. panamensis* strain MHOM/COL/81/L13 at a concentration of 1 × 10^7^ parasites/mL, freezing in liquid nitrogen and thawing at 37°C four times. Cells were suspended in RMPI 1640 (Sigma-Aldrich) with 10% FBS (Gibco, Carlsbad, CA), 2 mM L-glutamine, penicillin (100 U/mL) and streptomycin (100 mg/mL) and distributed in 96 well plates in 200 μL per well. For PBMC cultures, 4 × 10^5^ PBMCs and for B cell/CD4 T cell co-cultures, 2 × 10^5^ B cells or 2 × 10^5^ T cells or both cell types were plated per well. Then 8 μL of pLAg were added to the appropriate wells to reach a parasite:cell ratio of 0.2:1. The cells were incubated for 5 days at 37°C with 5% CO_2_. Cells and supernatants were harvested for evaluation of cell surface markers and cytokine secretion, respectively.

Ramos cells were cultured in RPMI 1640 with 10% heat-inactivated FBS at a concentration of 10^6^ cells/mL with pLAg (10^7^ parasites/mL final concentration), 5 μM CpG ODN 2006 (InvivoGen, San Diego, CA) or no stimulus for 48 hours. Cells were harvested for evaluation of cell surface markers or BCR-mediated endocytosis.

### Endocytosis assay

Ramos cells were incubated with goat F(ab’)_2_ anti-human IgM-biotin (Invitrogen, Camarillo, CA) at 4°C for 20 minutes, washed twice with PBS, resuspended in media and incubated with the Ova Antigen Delivery Reagent (Ova-FITC conjugated to an anti-biotin antibody, Miltenyi Biotec, Bergisch-Gladbach, Germany) at 37°C for different time periods, at which cells were harvested and fluorescence evaluated by flow cytometry.

### Flow cytometry

The following antibodies were used for cell surface marker evaluation: anti-CD4-PE-Cy5, anti-CD69-PE, anti-CD127-PE-Cy7, anti-CD20-PE-Cy7, anti-CD86-PE, anti-CD80-FITC (eBioscience, San Diego, CA), anti-CD25-ECD, anti-HLA-DR-ECD, and anti-CD11c-PC5 (Beckman Coulter, Brea, CA). Primary human cells or Ramos cells were suspended in FACS buffer (1× PBS, 1% BSA, 0.1% NaN_3_) and incubated for 20 minutes with panels of the indicated antibodies at 4°C. After washing, cells were analyzed in a Navios flow cytometer (Beckman Coulter, Brea, CA) and data was analyzed using FlowJo 7.6 software (Tree Star, Inc., Ashland, OR). Gates used for analysis were set using appropriate isotype controls.

### Cytokine measurement

Cytokines were measured in culture supernatants using the Human Th1/Th2/Th9/Th17/Th22 13plex FlowCytomix Multiplex (eBioscience) following the manufacturer’s instructions. Data were analyzed using FlowCytomix Pro Software (eBioscience).

### Statistical analysis

The Kolmogorov-Smirnov test was used to determine parametric or non-parametric distribution of the data. Thereafter, for comparisons between healthy subjects and CL patients, parametric data were analyzed using student t-test, and the Mann–Whitney test was applied for non-parametric data. For comparisons between CL patient cell cultures with *L. panamensis*- and control cultures, parametric and non-parametric data were analyzed using the paired t-test and the Wilcoxon signed-rank test, respectively. Statistical significance was defined as p < 0.05. All data were analyzed using Prism 5 software (GraphPad Software, Inc., La Jolla, CA).

## Results and discussion

### CD4 T cells and B cells of CL patients are concomitantly activated by *L. panamensis* in PBMC cultures

**Figure 1:**
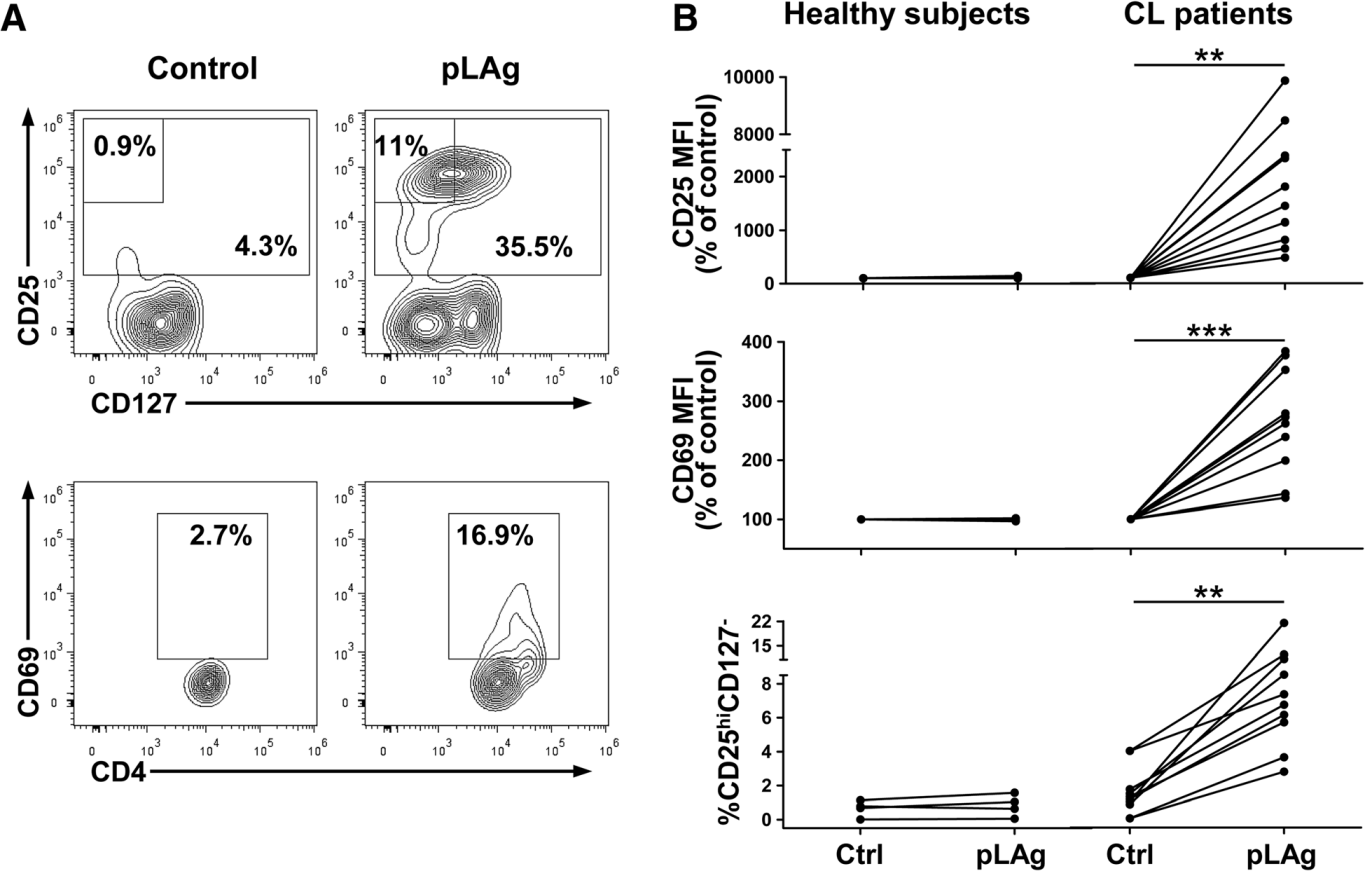
**Stimulation with pLAg induces upregulation of activation/regulatory markers in CD4 T cells from CL patients.** PBMCs were isolated from peripheral blood of CL patients (n = 10) and healthy subjects (n = 4), incubated with promastigote *L. panamensis* antigen (pLAg) for 5 days and stained for CD4, CD25, CD127, and CD69. **A**. Contour plots of cells within the CD4^+^ lymphocyte gate from one representative CL patient showing the effects of pLAg on the expression of CD25, CD127, and CD69. **B**. Expression of CD25 and CD69, and percentage of CD25^hi^CD127^-^ cells within the CD4^+^ gate for healthy subjects and CL patients. **p < 0.01.

We have previously shown that *L. panamensis* antigens induce CD4 T cell proliferation and cytokine secretion *in vitro* in CL patients and that immune response profiles differ in relation with clinical outcome; further, responses are specific in that no response to pLAg is observed with cells from uninfected individuals [5,38,40]. To determine whether B cells contribute to CD4 T cell activation in the context of active disease, we evaluated the activation of both CD4 T cells and B cells by *Leishmania* antigens in PBMC cultures. We found that the activation markers CD25 and CD69 were upregulated in CD4^+^ lymphocytes after incubation with pLAg (Figure 1A and B) and that CD4 T cells with the CD25^hi^CD127^-^ regulatory phenotype were induced by re-exposure to parasite antigens *in vitro* (Figure 1A and B). We have previously shown that cells with this regulatory phenotype are increased after treatment of chronic CL in patients from this region [38]. PBMCs from healthy subjects exposed to pLAg showed no activation marker upregulation or increase in the frequency of CD25^hi^CD127^-^ cells (Figure 1B). Thus, the changes in activation/regulatory markers observed in cells from CL patients likely represent the re-activation or expansion of cells specific for *Leishmania* antigens that were primed *in vivo*.

**Figure 2:**
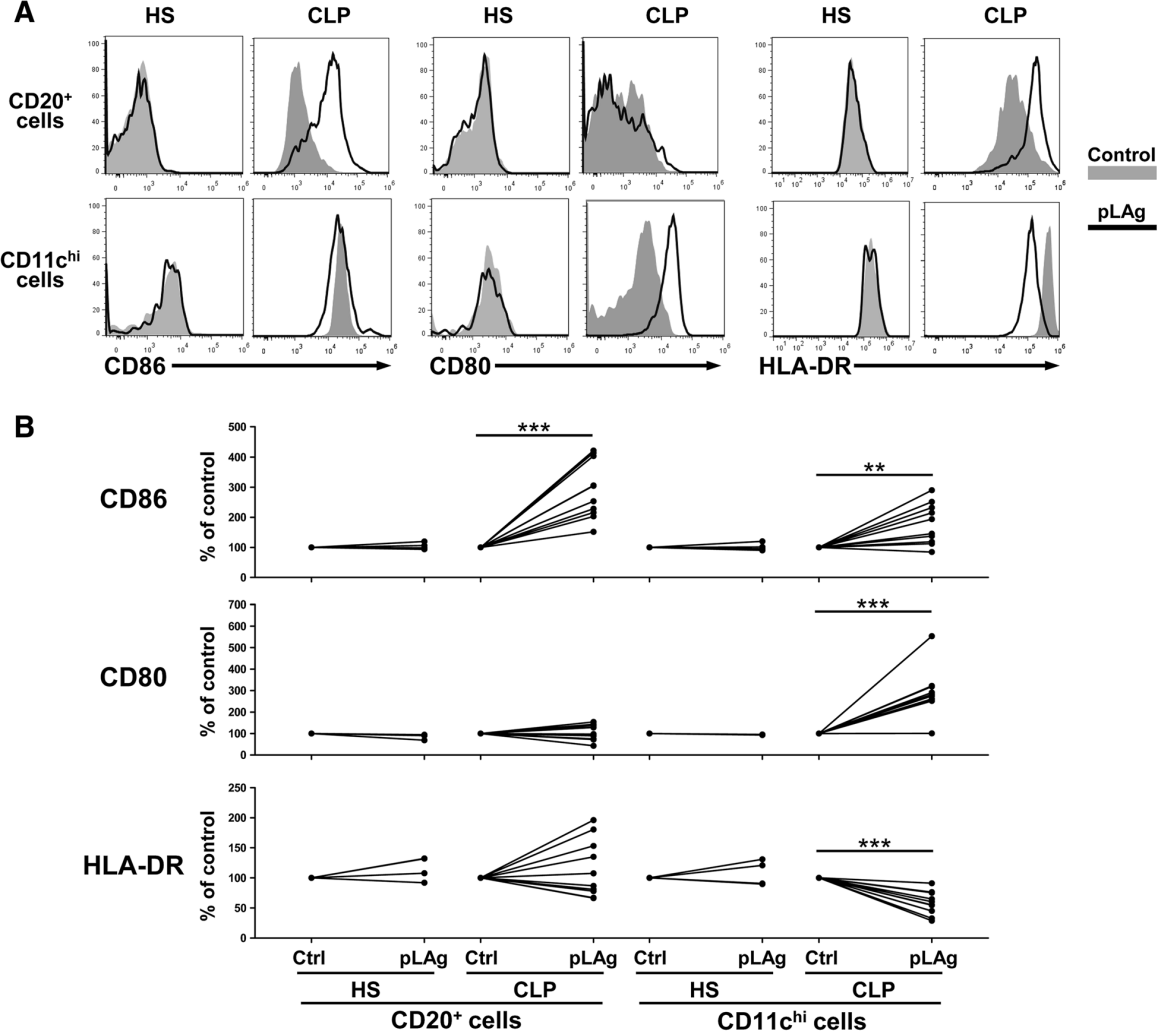
**Stimulation with pLAg induces upregulation of costimulatory molecules in APCs from CL patients.** PBMCs were isolated from peripheral blood of CL patients (CLP, n = 10) and healthy subjects (HS, n = 4), incubated with promastigote *L. panamensis* antigen (pLAg) for 5 days and stained for CD20, CD11c, CD86, CD80 and HLA-DR. **A**. Histograms of cells within the CD20^+^ lymphocyte gate (B cells, top panels) and CD11c^hi^FS^hi^ gate (DCs, bottom panels) from representative HS and CLP showing the effects of pLAg on expression of CD86, CD80 and HLA-DR. **B**. Percentages of variation in mean fluorescence intensity (MFI) of each marker in cells incubated with pLAg in relation to controls for HS and CLP. **p < 0.01, ***p < 0.001.

To evaluate the potential of B cells as APCs in these cultures, we evaluated the expression of the costimulatory molecules CD86 and CD80 and MHCII molecules (HLA-DR) in B cells (identified as CD20^+^ lymphocytes) compared to DCs (identified as CD11c^hi^FS^hi^ cells), recognized APCs and potent inducers of the adaptive immune response. As shown in Figure 2A, after 5 days of culture without antigen, DCs showed higher expression of CD86 and HLA-DR than B cells. However, when incubated with pLAg, B cells showed strong upregulation of CD86, while CD80 and HLA-DR underwent no significant changes. Meanwhile, DCs upregulated CD86 to a lesser extent and showed strong upregulation of CD80, but HLA-DR was downregulated (Figure 2). Exposure of cells from healthy subjects to pLAg did not result in significant changes, indicating that priming of cells *in vivo* was necessary for the observed activation (Figure 2). These results show that B cells specific for *Leishmania* antigens present in the blood of CL patients are able to attain the costimulatory activities needed for antigen presentation while maintaining MHCII expression. DCs upregulated costimulatory molecules but presented a significant decrease of MHCII expression after interaction with *L. panamensis* antigens. Even though several reports show downregulation of MHCII molecules after *Leishmania* infection in both DCs [18,41,42] and macrophages [10,13,43], we are not aware of any studies showing MHCII downregulation in DCs after incubation with soluble *Leishmania* antigens. Despite this decrease of MHCII, high expression of these molecules was still evident in DCs, indicating that this cell type was also poised to be a competent APC. Since both CD80 and CD86 have been shown to be able to provide the costimulation needed for CD4 T cell activation [44,45], these results allow us to conclude that in these cultures both B cells and DCs had a profile consistent with APC activation.

**Figure 3:**
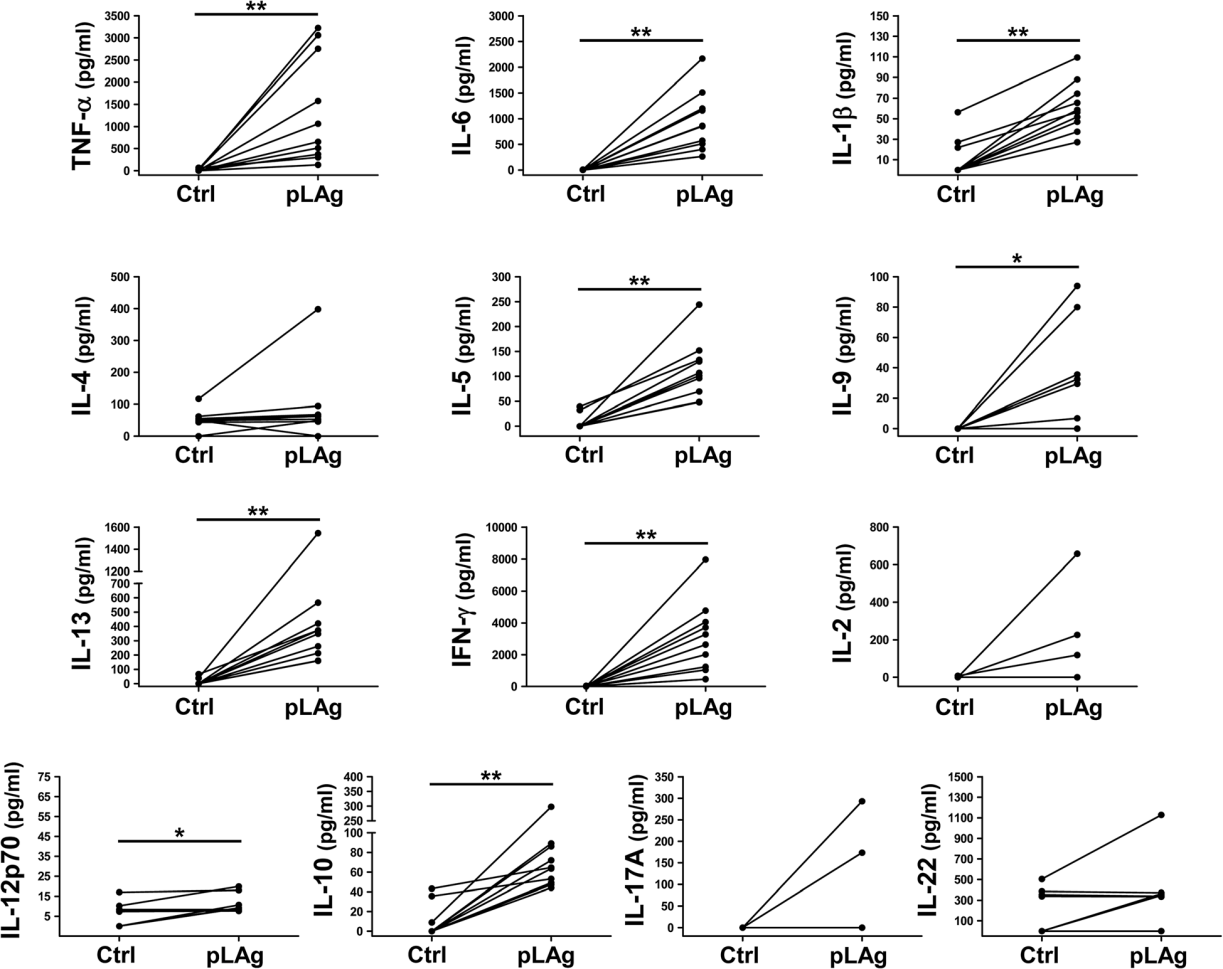
**Stimulation with pLAg induces cytokine secretion in PBMCs from CL patients.** PBMCs were isolated from peripheral blood of CL patients (n = 10), incubated with promastigote *L. panamensis* antigen (pLAg) for 5 days and the indicated cytokines were measured in supernatants with a bead-based assay. *p < 0.05, **p < 0.01.

To establish the cytokine profile induced by *L. panamensis* in PBMCs from CL patients, we measured 13 cytokines in culture supernatants. We found that incubation with pLAg induced significant levels of IFN-γ, TNF-α, IL-13 and IL-6 (Figure 3). The cytokines IL-10, IL-12p70, IL-1β, IL-5 and IL-9 were secreted in lower quantities, yet the increase was statistically significant in relation to controls. No significant production of IL-2, IL-17A, IL-4 or IL-22 was induced (Figure 3). This cytokine profile, which includes pro-inflammatory, Th1, Th2 and regulatory cytokines, is consistent with previous studies of patients from this region [4-8].

### Purified B cells are capable of activating CD4 T cells and inducing cells with a regulatory phenotype in the absence of other APCs

**Figure 4:**
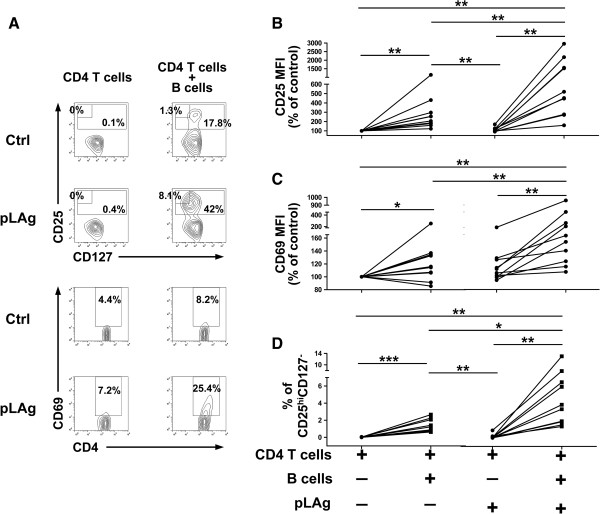
**Purified B cells induce CD4 T cell activation in CL patients.** B cells and CD4 T cells were purified from PBMCs of CL patients (n = 10), incubated for 5 days separately or together, with or without promastigote *L. panamensis* antigen (pLAg), and stained for CD4, CD25, CD127, and CD69. **A**. Representative plots for each culture condition showing the expression of CD25, CD127 and CD69 in the CD4^+^ gate. **B**. Expression of CD25 within the CD4^+^ gate. **C**. Expression of CD69 within the CD4^+^ gate. **D**. Percentage of CD25^hi^CD127^-^ cells within the CD4^+^ gate. *p < 0.05, **p < 0.01, ***p < 0.001.

The parallel activation of antigen-specific B cells and CD4 T cells in CL patients suggested that B cells may be able to induce CD4 T cell activation. To test this hypothesis, B cells and CD4 T cells purified from PBMCs of CL patients were stimulated with pLAg. CD25 and CD69 expression were significantly increased in CD4 T cells when incubated with B cells and pLAg in relation to cells cultured without antigen, or CD4 T cells incubated in the absence of B cells, with or without pLAg (Figure 4A, B and C). Although a trend of increased CD69 expression was observed for naïve T cells in the presence of pLAg, this increase was not significant (p = 0.08). Induction of CD25^hi^CD127^-^ CD4 T cells was also observed after incubation with B cells and pLAg and was not evident when either antigen or B cells were absent from cultures (Figure 4A and D). A paired analysis of the net induction of the evaluated CD4 T cell activation parameters in PBMC or purified B cell/CD4 T cell cultures did not reveal statistically significant differences, corroborating the comparable activation of CD4 T cells by *Leishmania* specific B cells and PBMCs from CL patients (data not shown).

**Figure 5:**
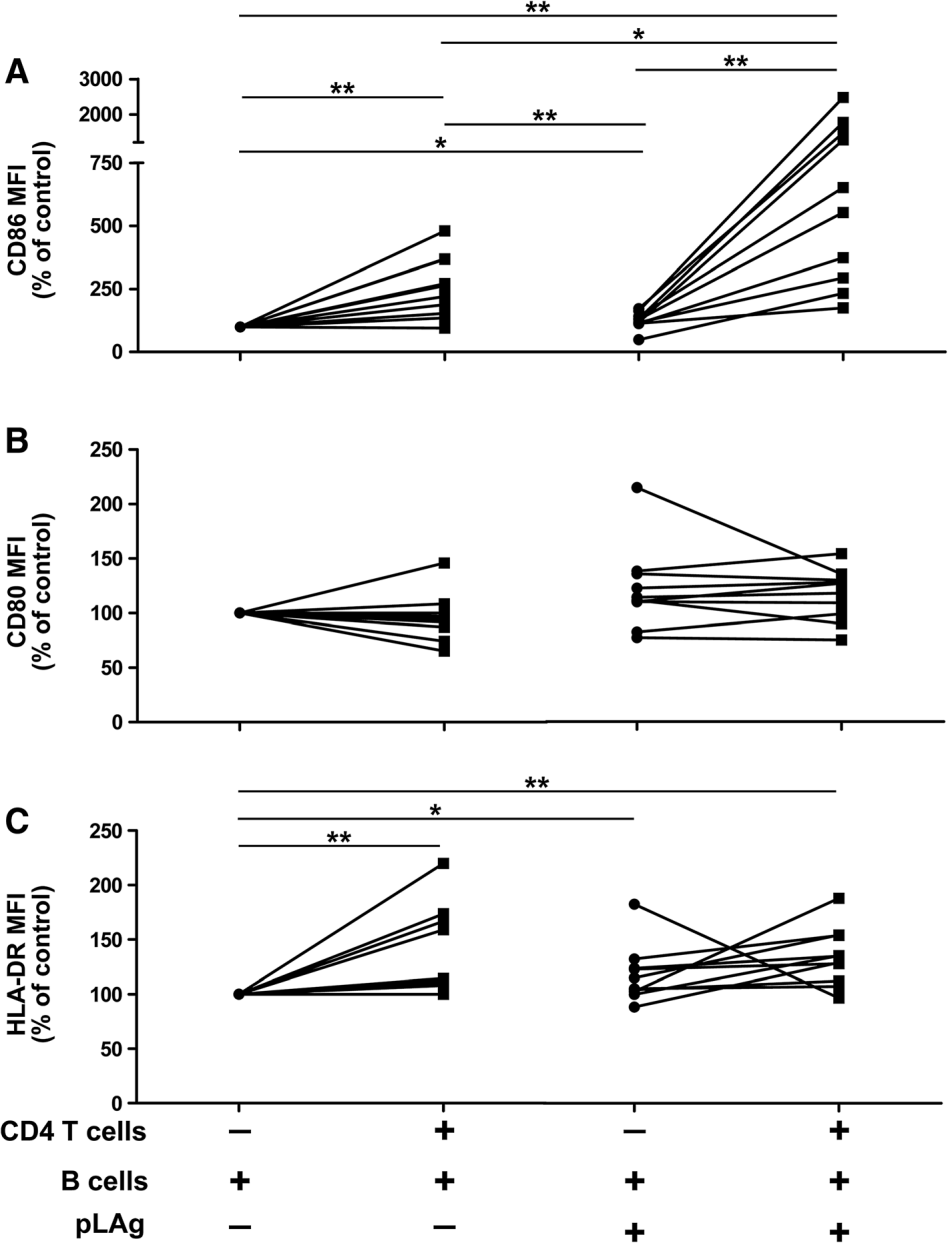
**B cells upregulate CD86 in the presence of CD4 T cells.** B cells and CD4 T cells were purified from PBMCs of CL patients (n = 10), incubated for 5 days separately or together, with or without promastigote *L. panamensis* antigen (pLAg), and stained for CD20, CD86, CD80, and HLA-DR. **A**. Expression of CD86 within the CD20^+^ gate. **B**. Expression of CD80 within the CD20^+^ gate. **C**. Expression of HLA-DR within the CD20^+^ gate. *p < 0.05, **p < 0.01.

**Figure 6:**
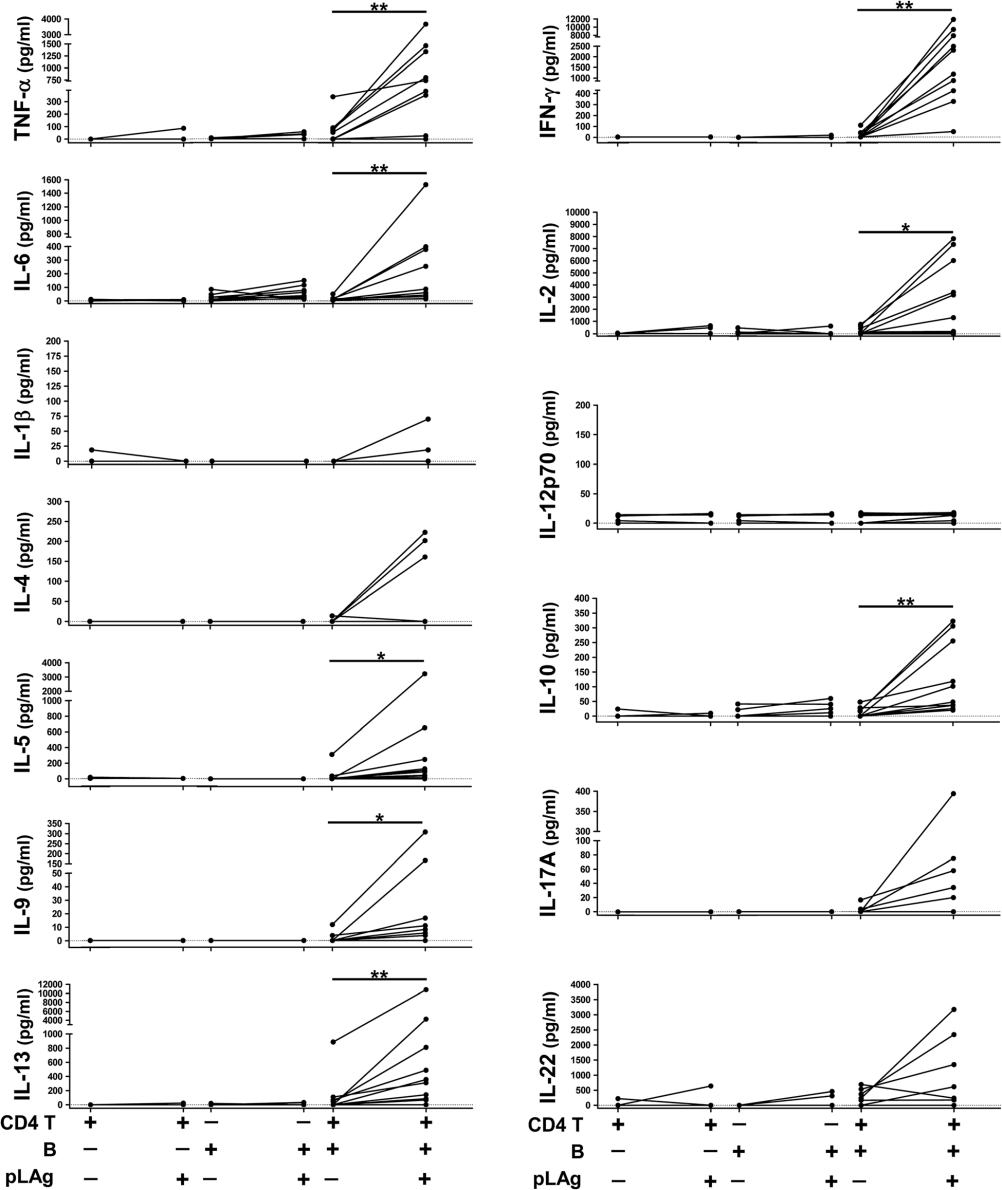
**Stimulation with pLAg induces cytokine secretion in B cell/CD4 T cell co-cultures from CL patients.** B cells and CD4 T cells were purified from PBMCs of CL patients (n = 10), incubated for 5 days separately or together, with or without promastigote *L. panamensis* antigen (pLAg) and the indicated cytokines were measured in supernatants with a bead-based assay. *p < 0.05, **p < 0.01.

To evaluate B cell activation in the B cell/CD4 T cell co-cultures, we analyzed CD86, CD80 and HLA-DR expression. pLAg did not activate B cells in the absence of CD4 T cells, but strong CD86 upregulation was induced when CD4 T cells and pLAg were present (Figure 5A). In concurrence with the PBMC cultures, addition of pLAg to purified B cell/CD4 T cells did not result in CD80 or HLA-DR upregulation (Figure 5B and C). These results demonstrate that to become competent CD4 activating cells, B cells require the presence of CD4 T cells. This likely reflects the need for costimulatory ligands expressed by activated CD4 T cells such as CD154 and cytokines [31,32]. We also determined the cytokine profile induced by *L. panamensis* antigens in B cell/CD4 T cell co-cultures. pLAg induced significant secretion of IFN-γ, TNF-α, IL-6, IL-13, IL-5, IL-9, IL-10 and IL-2, while no significant amounts of IL-12p70, IL-1β, IL-17A, IL-4 or IL-22 were detected (Figure 6). Importantly, no significant secretion of any cytokine was observed when either CD4 T cells or B cells were incubated alone with pLAg (Figure 6).

These results demonstrate that the capacity of isolated B cells from CL patients to activate CD4 T cells is similar to that of all APCs present in PBMCs. We performed a paired analysis to compare the net increase for each CD4 T cell activation parameter between PBMC and B cell/CD4 T cell cultures and found no significant differences. Likewise, net cytokine secretion did not reveal significant differences except for IL-12p70 and IL-1β (which were secreted in low albeit significant amounts only in PBMC cultures), IL-6 (which was secreted in both types of cultures but in significant larger amounts in PBMC cultures) and IL-2 (which was only secreted in B cell/CD4 T cell cultures). Thus, 10 of the 13 cytokines measured had similar levels in both types of cultures in spite of the fact that PBMCs contain several cytokine-producing cell types besides B cells and CD4 T cells. The differences observed in IL-12p70, IL-1β and IL-6 can be explained by the presence of mononuclear phagocytes in PBMCs that are known sources of these cytokines. Significant IL-2 production only in B cell/CD4 T cell cultures suggests that secretion of this cytokine is efficiently induced by interaction of these cell types.

The known mechanisms for CD4 T cell activation by T cells are antigen presentation and cytokine secretion. Since B cells stimulated with pLAg did not produce any significant amount of 13 cytokines that are prominent in antigen induced recall responses, our data strongly suggest that presentation of *L. panamensis* antigens by B cells is responsible for CD4 T cell activation. B cells can encounter soluble antigen soon after its entry into the body by several mechanisms and internalize it efficiently by BCR-mediated endocytosis [46]. Therefore, the fact that B cells are not phagocytic cells, and thus not a natural cellular host for *Leishmania*, does not diminish the relevance of their CD4 T cell activation function in CL. On the contrary, this characteristic may be crucial for their role as APCs in this disease since processing and presentation of particulate antigens on MHCII requires phagosome maturation, a process that can be inhibited by *Leishmania*[47], whereas presentation of soluble antigens on MHCII requires their correct targeting by the endocytic receptor, a process that is highly efficient after BCR-mediated endocytosis [30,48]. Accordingly, a study by Carvalho et al. found that DCs that were infected by *L. braziliensis* did not upregulate costimulatory and MHCII molecules, while bystander DCs were activated and presented soluble antigen efficiently [18]. An earlier study by Fruth et al. also showed that infection of macrophages with *L. major* inhibited APC function, but pulsing with *Leishmania* antigens did not [9]. Consequently, since infection of APCs with live parasites inhibits their function, APCs such as B cells that do not harbor the parasite and are capable of handling soluble antigens may be ideally poised to activate the immune response.

Cultures of purified lymphocytes from CL patients also showed a small but significant increase in the expression of CD25 and CD69 and in the frequency of CD25^hi^CD127^-^ cells among CD4 T cells when these cells were co-cultured with B cells in the absence of pLAg (Figure 4B-D). We also found a significant induction of CD86 and HLA-DR in B cells under the same conditions (Figure 5A and C). These results suggest that non-cognate interactions between B cells and CD4 T cells can generate activating signals in this setting. The induction of homeostatic lymphocyte proliferation by MHCII/self-peptide presented by APCs is well documented [32,49,50]. Hence, the activation observed in B cell/CD4T cell cultures in the absence of antigen may represent this type of interaction. Alternatively, the activation found in the absence of added antigen may be due to B cells from infected individuals already being loaded with *Leishmania* antigens, since we have found evidence of parasites in blood, during active CL and even after treatment of the disease [51].

### Treatment with *L. panamensis* upregulates MHCII and costimualtory molecules independently of BCR signaling and enhances BCR-mediated endocytosis in human B cells

**Figure 7:**
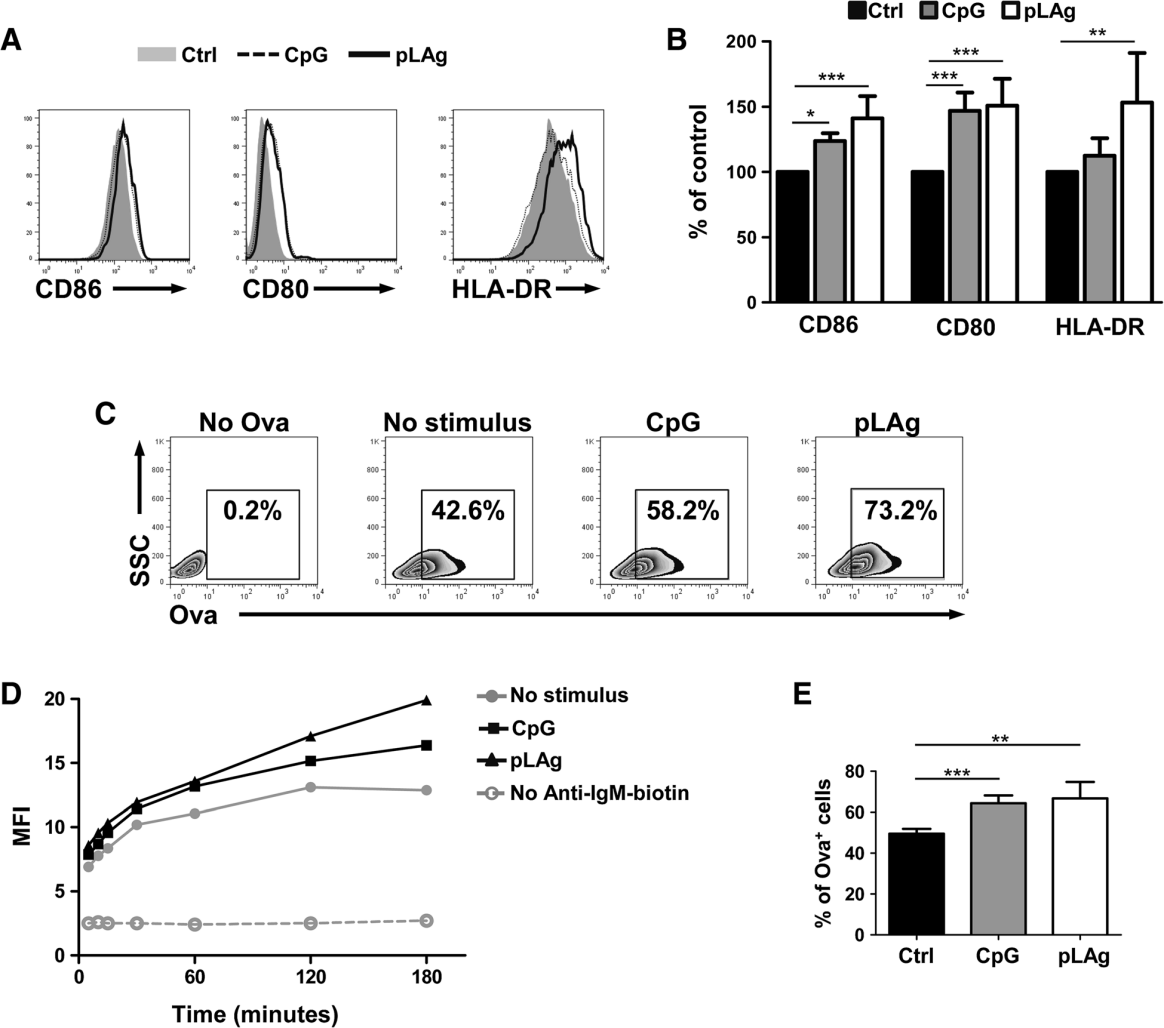
***L. panamensis *upregulates costimulatory and MHC molecules and enhances BCR-mediated endocytosis in Ramos cells.** Ramos cells were cultured without stimulus or treated with CpG or promastigote *L. panamensis* antigen (pLAg) for 48 hours and then incubated with anti-IgM-biotin at 4°C and Ova-FITC-anti-biotin at 37°C for up to 3 hours. **A**. Expression of CD86, CD80 and HLA-DR after 48 hours of treatment. **B**. Means ± SD of four experiments are shown. **C**. FACS analysis of the frequency of Ova-FITC positive cells after 1 hour of endocytosis. Representative plots for one experiment are shown. **D**. Mean fluorescence intensity (MFI) for Ova-FITC in Ramos cells after 5, 10, 15, 30, 60, 120 and 180 minutes of endocytosis. One representative experiment of two is shown. **E**. Frequency of cells positive for Ova-FITC after 1 hour of endocytosis. Means ± SD of four experiments are shown. *p < 0.05; **p < 0.01; ***p < 0.001.

BCR mediated endocytosis is one of the key processes that allow B cells to become potent APCs. Therefore, we next evaluated whether *L. panamensis* antigen enhances this process. For this purpose, we used a model in which both antigen and B cells would interact in a uniform manner. Since the number and specificity of B cells primed by *Leishmania* infection *in vivo* are uncertain, we did not use primary cells from CL patients. Rather, we used Ramos cells, a human B cell line derived from a lymphoma that expresses surface IgM not specific for *Leishmania* antigens. We first evaluated if upregulation of costimulatory and MHC molecules could be achieved after treating Ramos cells with pLAg. Because Ramos cells express TLR9, we used CpG as a positive control of a BCR-independent activation signal. We found that the pLAg at the 0.2 parasites:1 cell ratio we used in cultures of primary cells did not induce a detectable response (data not shown). To detect a significant effect, we had to increase the parasite antigen concentration 50-fold. At a ratio of 10 parasites:1 cell, increased expression of CD86, CD80 and HLA-DR was observed after 48 hours of culture to levels comparable to those induced by CpG (Figure 7A and B). Thus, *L. panamensis* antigen can induce upregulation of activation markers in Ramos cells without BCR engagement.

To test the effect of *L. panamensis* antigen on BCR-mediated endocytosis in Ramos cells, we targeted the model antigen ovalbumin (Ova) to the BCR using an anti-IgM antibody. BCR-mediated endocytosis of fluorescent Ova was evaluated in cells previously cultured for 48 hours with pLAg or CpG or without stimulus, as described in Materials and Methods. We found that Ova endocytosis by Ramos cells increased over the first two hours and then reached a plateau. When these cells were treated with CpG or pLAg, Ova endocytosis was more efficient and continued to increase throughout the period of evaluation (Figure 7C and D). A statistically significant difference was detected for both pLAg and CpG when compared to untreated cells both with respect to fluorescence intensity and the percentage of Ova-FITC positive cells at the 1 hour time point (Figure 7E). When OVA antigen was not targeted to the BCR, fluorescence did not increase at any time point, confirming that the assay evaluated BCR-mediated endocytosis (Figure 7D). This experimental strategy allowed us to show that *L. panamensis* antigen promotes specific antigen uptake by human B cells. The expression of surface IgM did not change significantly after treatment with CpG or pLAg (data not shown), indicating that the enhanced endocytosis was not due to the availability of more internalizing receptors. Similar enhancement of Ova endocytosis was previously observed in DCs incubated with *L. braziliensis* antigens for four days [18].

These results demonstrate that *L. panamensis* antigen can induce activation of B cell antigen presenting function without BCR engagement. TLR9 is the innate immune receptor that has the highest expression on human B cells [52-54] and it can be stimulated by *Leishmania* DNA [55,56]. Since the pLAg preparation includes the parasite DNA, it is likely that TLR9 signaling can mediate activation of the antigen presenting function of human B cells, a question that we will address in future studies. In humans, B cells and plasmacytoid DCs are the only cell types responsive to TLR9 [57,58]. Because the antigen presenting capacity of plasmacytoid DCs is low [59] and is further downregulated by TLR9 signaling [60], B cells constitute the primary APC targeted by TLR9 ligands in humans. In light of the numerous examples of successful protection in animals using CpG as adjuvant in vaccination strategies for CL [61-64], defining the role of TLR9 in B cell antigen presentation is critical to develop efficient strategies that can be translated into clinical applications.

## Conclusions

We have shown that B cells from the peripheral blood of CL patients are capable of inducing CD4 T cell activation and cytokine secretion, an effect likely mediated by antigen presentation. The CD4 T cell activating capacity of these B cells was similar in both quantity and quality to that observed after incubation with all cells present in PBMCs. These B cells represent an attractive target for immunomodulatory strategies that aim to redirect the host’s immune response to a healing phenotype in chronic *Leishmania (Viannia)* infections.

## Abbreviations

CL: Cutaneous leishmaniasis; APCs: Professional antigen presenting cells; DCs: Dendritic cells; PBMCs: Peripheral blood mononuclear cells; pLAg: Promastigote *L. panamensis* antigen.

## Competing interests

The authors declare that they have no competing interests.

## Authors’ contributions

All authors participated in conception and design of the study and data interpretation. DR-P performed the experiments and wrote the manuscript. NGS and DM-P critically reviewed and and contributed to the writing of the manuscript. All authors reviewed and approved the final version of the manuscript.

## Authors’ information

Dr. Rodriguez-Pinto’s current address: Facultad de Medicina, Facultad de Ciencias de la Salud, Universidad de las Américas, Quito, Ecuador.

## Pre-publication history

The pre-publication history for this paper can be accessed here:

http://www.biomedcentral.com/1471-2334/14/108/prepub
